# Gold-patched graphene nano-stripes for high-responsivity and ultrafast photodetection from the visible to infrared regime

**DOI:** 10.1038/s41377-018-0020-2

**Published:** 2018-06-20

**Authors:** Semih Cakmakyapan, Ping Keng Lu, Aryan Navabi, Mona Jarrahi

**Affiliations:** Electrical Engineering Department, University of California Los Angeles, Los Angeles, CA 90095 USA

## Abstract

Graphene is a very attractive material for broadband photodetection in hyperspectral imaging and sensing systems. However, its potential use has been hindered by tradeoffs between the responsivity, bandwidth, and operation speed of existing graphene photodetectors. Here, we present engineered photoconductive nanostructures based on gold-patched graphene nano-stripes, which enable simultaneous broadband and ultrafast photodetection with high responsivity. These nanostructures merge the advantages of broadband optical absorption, ultrafast photocarrier transport, and carrier multiplication within graphene nano-stripes with the ultrafast transport of photocarriers to gold patches before recombination. Through this approach, high-responsivity operation is realized without the use of bandwidth-limiting and speed-limiting quantum dots, defect states, or tunneling barriers. We demonstrate high-responsivity photodetection from the visible to infrared regime (0.6 A/W at 0.8 μm and 11.5 A/W at 20 μm), with operation speeds exceeding 50 GHz. Our results demonstrate improvement of the response times by more than seven orders of magnitude and an increase in bandwidths of one order of magnitude compared to those of higher-responsivity graphene photodetectors based on quantum dots and tunneling barriers.

## Materials: Gold-patched graphene nano-stripes pave way for broadband and ultrafast photodetectors

Scientists have made graphene nano-stripes that could lead to photodetector technologies with greater bandwidth, ultrafast speed, and high  responsivity for use in imaging and sensing systems. Graphene has become an attractive material for use in broadband photodetection technologies owing to its unique electronic and optical properties, such as its high electrical conductivity and ability to absorb a broad spectral range of light. However, its potential has been hindered by tradeoffs between the responsivity, bandwidth and speed of existing graphene photodetectors. Now, Mona Jarrahi and colleagues from the University of California Los Angeles, in America, have fabricated gold-patched graphene nano-stripes that offer broadband, ultrafast, and highly responsive photodetection. The work has led to photodetectors with response times more than seven orders of magnitude, and bandwidths of one order of magnitude greater than currently available.

## Introduction

Graphene has rapidly become an attractive candidate material for broadband and ultrafast photodetection because of its distinct optical and electronic characteristics^1,2^. These characteristics stem from the unique band structure of graphene, which enables carrier generation via optical absorption over an extremely broad spectral range from the ultraviolet to microwave regime. Moreover, the high electron/hole mobility and weak scattering in graphene enables an ultrafast temporal response in graphene photodetectors. Additionally, the two-dimensional nature of graphene enables multiple electron/hole pairs to be generated for a single absorbed photon^3–5^. Furthermore, the compatibility of graphene photodetectors with silicon-based fabrication platforms enables integration with low-cost and high-performance complementary metal oxide semiconductor read-out and post-processing circuits.

Most graphene photodetectors utilize graphene–metal junctions or graphene p–n junctions to spatially separate and extract photogenerated carriers. The low optical absorption inside the effective junction regions (~100−200 nm) and short photocarrier lifetime of graphene (~1 ps) represent the two major challenges for the development of high-responsivity graphene photodetectors^6^. Various techniques have been explored to address these challenges and to enhance the responsivity of graphene photodetectors. Despite the significant advantages of these techniques for offering high photodetection responsivities, the scope and potential use of existing graphene photodetectors remain limited by tradeoffs between high responsivity, an ultrafast temporal response, and broadband operation.

For example, responsivity-enhanced photodetection in graphene from the visible to mid-infrared wavelengths has been achieved by increasing the photocarrier lifetime through band-structure engineering and defect engineering. For this purpose, carrier trapping mechanisms and patterned graphene nanostructures have been utilized to introduce bandgap and midgap defect states in graphene^7–10^. However, the response times for these graphene photodetectors have been limited by long carrier trapping times due to the introduced defect and edge states. Hybrid graphene–quantum dot photodetectors offer a powerful alternative for enhancing the photodetection responsivity by increasing light absorption and introducing large carrier multiplication factors^11–14^. However, the bandwidth and response time for this type of graphene photodetector are restricted by the narrow spectral bandwidth and long carrier trapping times of the quantum dots. Photodetectors based on two graphene layers separated by a thin tunnel barrier have also led to enhanced broadband responsivity via separation of the photogenerated electrons and holes through quantum tunneling and minimization of their recombination^15^. However, the response times for this type of graphene photodetector have been limited by the long carrier trapping times in the tunneling barriers utilized. Waveguide-integrated graphene photodetectors have been considered to be another promising alternative and offer enhanced ultrafast responsivity by increasing the interaction length of light within graphene^16–19^. These graphene photodetectors have the additional advantage of process compatibility with standard photonic integrated circuits. However, their spectral bandwidth has been restricted by the bandwidth limitations of the waveguides utilized. Moreover, microcavities, plasmonic structures, and optical antennae have all been integrated with graphene to achieve high responsivities by increasing the interaction length of light within graphene^20–32^. However, the bandwidth of these types of graphene photodetectors has been limited by the resonant nature of the structures utilized.

In this work, we use engineered photoconductive nanostructures based on gold-patched graphene nano-stripes, which have unique electrical and optical characteristics that enable simultaneous broadband and ultrafast photodetection with high responsivity. The key novelty that enables the superior performance of these photoconductive nanostructures is the confinement of most of the photocarrier generation and conduction to the graphene nano-stripes and gold patches, respectively. Therefore, such nanostructures benefit from the broadband optical absorption and photocarrier multiplication capabilities of graphene and avoid the negative effects of the short photocarrier lifetime of graphene.

## Materials and methods

### Device fabrication

Commercially available chemical vapor deposition (CVD)-grown graphene is first transferred to a high-resistivity silicon wafer covered with a 130-nm-thick thermally grown SiO_2_ layer. The gold patches are then patterned by electron beam lithography and formed by 5/45 nm Ti/Au deposition and liftoff. The graphene nano-stripes are then patterned by another electron beam lithography step and formed by oxygen plasma etching. Next, the bias lines and output pads are patterned by optical lithography and formed by 20/200 nm Ti/Au deposition and liftoff. Finally, gate pads are patterned by optical lithography and formed by SiO_2_ plasma etching, followed by 20/200 nm Ti/Au deposition and liftoff.

### Responsivity measurements

A supercontinuum laser (NKT Photonics, Portland, OR, USA; SuperK EXTREME) is used to measure the photodetector responsivity in the visible/near-infrared regime (0.8–1.8 μm wavelength range). The fiber-coupled output of the supercontinuum laser is placed very close to the device to ensure that all of the output power is incident on the device active area (30 × 30 μm^2^). The responsivity values are calculated from the photocurrent measured using a source measure unit (Keithley; 2450 SourceMeter), and the optical power measured using a calibrated near-infrared photodetector (Thorlabs; S132C). A Globar light source (Thorlabs; SLS203L) combined with different infrared bandpass filters is used to measure the photodetector responsivity in the infrared range (3–20 μm wavelength range). A list of the filters used, including their spectral characteristics, is given in Supplementary Table S1. A calibrated calorimeter (Scientech; AC2500S Calorimeter and Vector S310) is used to measure the infrared radiation at each wavelength. The calorimeter is positioned at a distance of 1 cm from the Globar output, where the infrared intensity is uniform across the calorimeter input aperture. The uniformity of the infrared beam is confirmed by replacing the calorimeter with a graphene photodetector and monitoring its output photocurrent while moving it in the plane normal to the incident beam. The responsivity values are calculated from the measured photocurrent and measured infrared power using the calorimeter and scaled according to the ratio between the active area of the graphene photodetector and calorimeter.

## Results and discussion

**Fig. 1:**
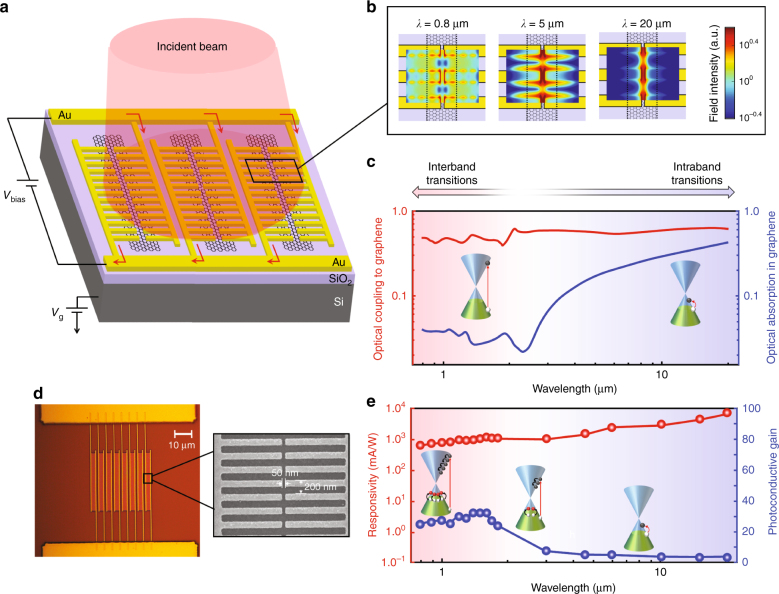
**High-responsivity and broadband photodetection via gold-patched graphene nano-stripes.** **a** Schematic of a photodetector based on gold-patched graphene nano-stripes. The photodetector is fabricated on a high-resistivity Si substrate coated with a 130-nm-thick SiO_2_ layer. The gate voltage applied to the Si substrate, *V*_g_, controls the Fermi energy level of the graphene nano-stripes. The gold patches have a width of 100 nm, periodicity of 200 nm, height of 50 nm, length of 1 μm, and a tip-to-tip gap size of 50 nm. **b** Color plot of the transmitted optical field, polarized normal to the graphene nano-stripes, through the gold patches at 0.8, 5, and 20 μm, indicating highly efficient and broadband optical coupling to the graphene nano-stripes. **c** Numerical estimates for the optical coupling (red curve) and optical absorption (blue curve) in the graphene nano-stripes as a function of wavelength. **d** Optical microscope and scanning electron microscopy (SEM) images for a fabricated photodetector based on gold-patched graphene nano-stripes. **e** The measured responsivity (red data) and photoconductive gain (blue data) of the fabricated photodetector at an optical power of 2.5 μW, gate voltage of 22 V, and bias voltage of 20 mV

The operation principle of the photodetector, which is based on utilization of the photoconductive nanostructures, is illustrated in Fig. 1a. Arrays of monolayer graphene nano-stripes are patterned onto a high-resistivity Si substrate ( > 10 kΩ·cm) coated with a 130-nm-thick thermal oxide. The carrier concentration and sheet resistance of the graphene nano-stripes used in this study are measured to be 1.8 × 10^13^ cm^−2^ and 804 Ω/□, respectively, when no gate bias is applied (Supplementary Fig. S1). The photoconductive nanostructures are formed by connecting arrays of nanoscale gold patches to either side of the graphene nano-stripes. The geometry of the gold patches is engineered to concentrate a major portion of the incident optical beam onto the graphene nano-stripes over a broad optical spectrum ranging from the visible to infrared regime. The graphene nano-stripes are designed to be narrower than the effective metal–graphene junction regions, where the photogenerated electrons and holes separate. This design enables a fast photocarrier transit time to the gold patches under an applied bias voltage because this transit time is much faster than the graphene photocarrier lifetime. Therefore, unlike previously demonstrated graphene photodetectors, which show high photoconductive gains via an increase in the photocarrier lifetime, the gold-patched graphene nano-stripes afford high photoconductive gains by reducing the photocarrier transport time to the gold patches. The photocarriers transported to the gold patches are all collected together (illustrated by red arrows in Fig. 1a) to form the output photocurrent of the photodetector.

Numerical finite difference time domain simulations (Lumerical) are carried out to demonstrate the unique capability of the designed gold patches to efficiently concentrate the incident optical beam onto the graphene nano-stripes over a broad optical wavelength range. Figure 1b shows a color plot of the transmitted optical field through the gold patches at 0.8, 5, and 20 μm for an incident optical beam polarized normal to the graphene nano-stripes, indicating highly efficient and broadband optical coupling to the graphene nano-stripes. The numerical analysis predicts that 40–60% of the incident optical beam can be focused onto the graphene nano-stripes over a broad spectrum ranging from the visible to infrared regime, as illustrated in Fig. 1c (red curve). Despite the broadband optical coupling to the graphene nano-stripes, the optical absorption in graphene is estimated to be highly wavelength dependent, as shown in Fig. 1c (blue curve). This strong wavelength dependence stems from the optical absorption in graphene being dominated by interband transitions in the visible and near-infrared spectral ranges and by intraband transitions in the infrared spectral range^33^, leading to much lower optical absorption in the visible and near-infrared regimes.

Gold-patched graphene nano-stripes can exploit the enhanced carrier multiexcitation generation that occurs at higher photon energy levels to compensate for the lower optical absorption at lower wavelengths^3–5^. Such carrier multiplication factors have not been previously exploited in monolayer graphene photodetectors without the use of quantum dots because of the short photocarrier lifetime in graphene^11,14^. However, carrier multiplication can be used to boost the photoconductive gain of the gold-patched graphene nano-stripes at lower wavelengths because of the fast photocarrier transport time to the gold patches. Since the use of any defect states and/or quantum dots is avoided, the gold-patched graphene nano-stripes enable high responsivity photodetection without sacrificing the ultrafast broadband operation.

Fig. 1d shows an optical microscope image for a fabricated photodetector based on gold-patched graphene nano-stripes; the photodetector has an active area of 30 × 30 μm^2^. This figure also shows a scanning electron microscopy image of the gold patches (for further details, see the Methods). A supercontinuum laser and Globar light source combined with bandpass filters are used to measure the photodetector responsivity in the visible/near-infrared and infrared regimes, respectively (for further details, see the Methods). The responsivity spectrum for the fabricated photodetector at an optical power of 2.5 μW, gate voltage of 25 V, and bias voltage of 20 mV is shown in Fig. 1e (red data). The photodetector has an ultrabroad operation bandwidth from the visible to the infrared regime with high-responsivity levels ranging from 0.6 A/W (at wavelength of 800 nm) to 8 A/W (at a wavelength of 20 μm). This graphene photodetector exhibits the widest photodetection bandwidth with the high responsivity reported to date, which are enabled by the use of the gold-patched graphene nano-stripes. In the following sections, we describe how even higher responsivity levels can be realized when the bias and gate voltages are optimized for the photodetector. In addition, the asymmetric geometry of the gold-patched graphene nano-stripes leads to a highly polarization-sensitive responsivity (Supplementary Fig. S2). This strong polarization sensitivity for the photodetector has many applications in polarimetric imaging and sensing systems.

The photoconductive gain of the fabricated photodetector is calculated from the measured responsivity and estimated optical absorption in graphene, as shown in Fig. 1e (blue data). As expected, higher photoconductive gains are obtained at lower wavelengths for which the photogenerated electrons are excited to higher energy levels in the conduction band. Excitation to higher energy levels gives rise to the excitation of secondary electron–hole pairs by transferring more energy during relaxation, as illustrated in the inset of Fig. 1e.

**Fig. 2:**
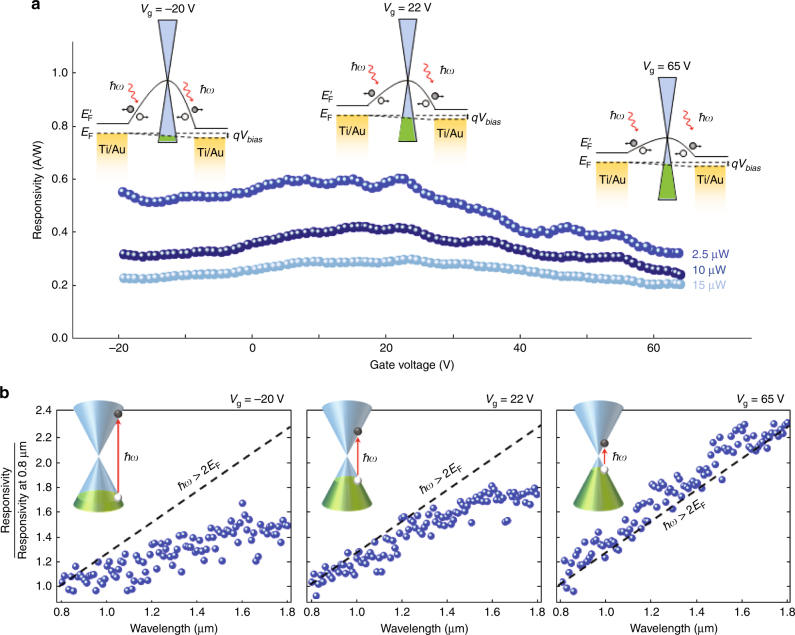
**Impact of the gate voltage on the photodetector performance in the visible and near-infrared regimes.** **a** Responsivity of the fabricated photodetector at a wavelength of 800 nm under a bias voltage of 20 mV. Inset figures show band diagrams for the graphene photodetector at gate voltages of −20, 22, and 65 V. The Fermi energy level (*E*_F_) and graphene Dirac point energy level (*E*_F_′) are illustrated by the dashed and solid black lines, respectively. The carrier concentration in the graphene nano-stripes used in this study is measured to be 1.8 × 10^13^ cm^−2^ when the gate is unbiased, indicating highly p-doped graphene nano-stripes at a gate voltage of −20 V. **b** Responsivity of the fabricated photodetector at optical wavelengths ranging from 800 nm to 1.8 μm and at gate voltages of −20, 22, and 65 V. The responsivity values at each gate voltage are divided by the photodetector responsivity at 800 nm to eliminate the influence of variations in the band diagram at different gate voltages. The dashed lines show the predicted responsivity spectra assuming interband transitions are allowed over the entire wavelength range

Fig. 2 shows the effect of the gate voltage on the photodetector responsivity in the visible and near-infrared regimes, where the optical absorption is dominated by interband transitions in graphene. The effect of the gate voltage is best described by the device band diagrams at various gate voltages (Fig. 2a inset), which illustrate that the gate voltage tunes the carrier concentration and Fermi energy level of the graphene nano-stripes between the gold patches while maintaining the same metal-induced doping levels at the gold-patch junctions. This tuning modifies the band-bending slope at different gate voltages (a detailed analysis of the device band diagram is included in Supplementary Fig. S3). When an optical beam is incident on the device, the photogenerated electrons and holes move according to the induced electric field determined by the band-bending slope. Therefore, the photogenerated holes move to the center of the graphene nano-stripes, where they eventually recombine, with the photogenerated electrons moving to the anode and cathode junctions. The induced photocurrent is proportional to the difference in the number of photogenerated electrons that drift to the anode and cathode junctions (Supplementary Fig. S4).

Figure 2a shows the responsivity of the fabricated photodetector at a wavelength of 800 nm and bias voltage of 20 mV when the gate voltage is varied between −20 and 65 V. As the gate voltage decreases from 65 V, the p-type carrier concentration in the graphene nano-stripes increases and a steeper energy band bending is introduced on the anode side compared to the cathode side^34,35^. The variation in the band-bending slopes leads to an increase in the induced photocurrent when the gate voltage decreases. This trend changes for gate voltages of <22 V, with a small decrease in the induced photocurrent observed for a further decrease of the gate voltage. The small reduction in the photocurrent at gate voltages <22 V can be explained by the decreased carrier mobility at high carrier densities due to the increase in carrier scattering^36–38^. The highest responsivity level is obtained at a gate voltage of 22 V. The photodetector responsivity decreases at higher optical powers (Supplementary Fig. S5). This decrease is due to the increase in the carrier recombination rate at high photogenerated carrier densities, which reduces the carrier scattering time and carrier multiplication efficiency^3–5^. Larger area gold-patched graphene nano-stripe arrays can be used to maintain high photodetection efficiencies at high optical powers. As expected, the measured photodetector responsivity shows a linear dependence on the applied bias voltage (Supplementary Fig. S6). This dependence suggests that higher responsivity levels can be realized by increasing the bias voltage at the expense of an increased dark current.

Figure 2b indicates how the gate voltage affects the photodetector responsivity by varying the optical absorption of graphene. The measured responsivity values in the visible and near-infrared regimes at gate voltages of −20, 22, and 65 V are shown. The responsivity values at each gate voltage are divided by the photodetector responsivity at 800 nm to eliminate the influence of variations in the band diagram at different gate voltages. Since the photodetector responsivity is proportional to the optical absorption coefficient divided by the photon energy, as far as optical absorption is governed by interband transitions in graphene, the photodetector responsivity should be linearly proportional to the photon wavelength (dashed line: responsivity/responsivity at 0.8 μm = *λ*/0.8 μm). However, because interband optical transitions are only allowed when *ħω≤*2*E*_f_, the photodetector responsivity is substantially reduced by Pauli blocking at lower photon energies when the gate voltage is lowered from 65 to 22 V and −20 V.

**Fig. 3:**
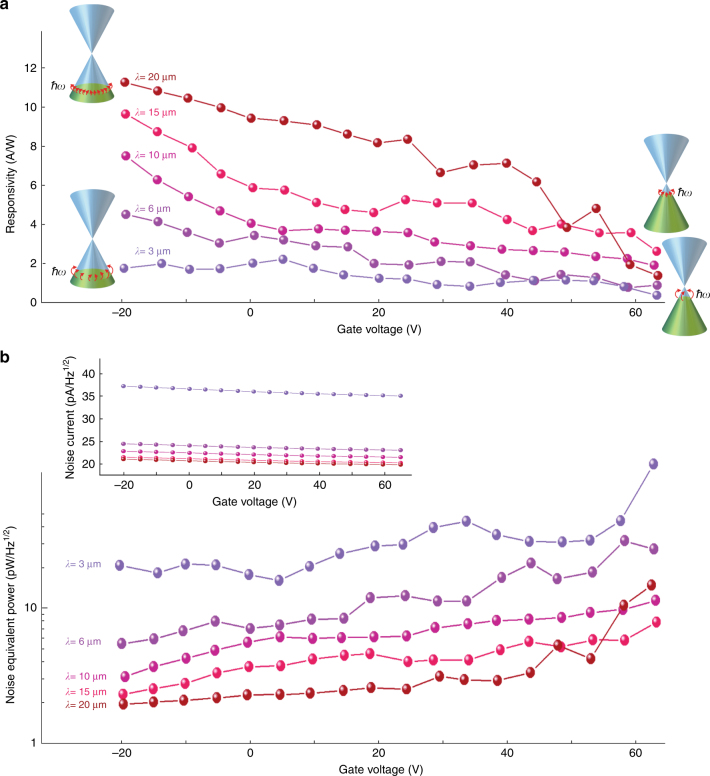
**Influence of the gate voltage on the photodetector performance in the infrared regime.** **a** Responsivity of the fabricated photodetector at wavelengths ranging from 3 to 20 μm. Inset figures illustrate intraband transitions at wavelengths of 3 and 20 μm under gate voltages of −20 and 65 V. The measured responsivity values show errors as large as ±20% because of thermal fluctuations in the measurement environment that lead to a ±20% variation in the output power of the Globar infrared source. **b** Noise equivalent power (NEP) for the fabricated photodetector at wavelengths ranging from 3 to 20 μm for an optical chopping frequency above 1 kHz. The inset shows the estimated noise current as a function of the gate voltage. All the measurements are carried out at a bias voltage of *V*_bias_ = 20 mV

Figure 3 shows the impact of the gate voltage on the photodetector performance in the infrared regime, in which the optical absorption is dominated by intraband transitions in the graphene. The impact of the gate voltage is best described by the graphene band diagrams at various gate voltages and wavelengths (Fig. 3a inset). These diagrams show that the gate voltage tunes the Fermi energy level, which changes the number of available states because of the cone-shaped band diagram of graphene. Since a larger number of states are available at higher Fermi energies, the photodetector responsivity values are increased following a decrease in the gate voltage at all wavelengths. At a given Fermi energy level, a larger number of states is available to be filled by lower-energy photons, which results in an increase in photodetector responsivity values at longer wavelengths. Responsivity values as high as 11.5 A/W are obtained at a wavelength of 20 μm wavelength and a gate voltage of −20 V, which correspond to the lowest photon energy and highest Fermi energy level in our measurements, respectively. Notably, the operation bandwidth of the photodetector is not limited to 20 μm, with higher responsivity values expected at longer wavelengths. The measurement bandwidth of our experimental setup was limited by the detection bandwidth of the calibrated infrared detector used for the measurements (for further details, see the Methods).

One of the drawbacks of the presented photodetector based on gold-patched graphene nano-stripes is the relatively large dark current that occurs due to the photoconductive nature of the photodetector. Therefore, the noise equivalent power (NEP) of the fabricated photodetector is calculated to assess the noise performance. For this calculation, we assume an optical chopping frequency above 1 kHz and lock-in the detection of the output signal to significantly reduce the 1/*f* noise current with respect to the Johnson Nyquist and shot-noise currents, which is similar to the operation of commercially available room-temperature infrared detectors^39,40^. Under this operation condition, the photodetector noise current, which is dominated by Johnson Nyquist and shot-noise sources, is extracted from the measured photocurrent and resistance data (Supplementary Fig. S7). The calculated NEP levels from the extracted noise current and measured responsivity values range from 1 to 20 pW/Hz^1/2^ (Fig. 3b), demonstrating superior noise performance compared to commercially available room-temperature infrared detectors under similar operation conditions^39,40^.

**Fig. 4:**
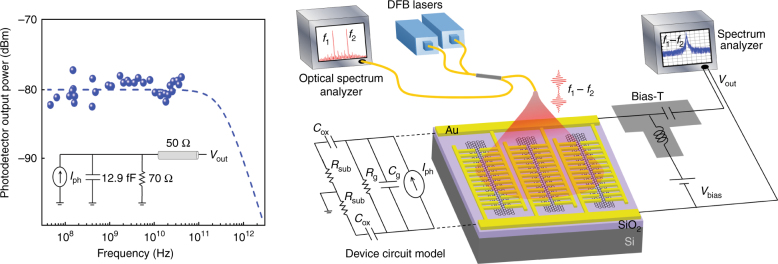
**Characterization of the operation speed of the fabricated photodetector.** The beams from two wavelength-tunable DFB lasers at frequencies of *f*_1_ and *f*_2_ are focused onto the gold-patched graphene nano-stripes to generate a photocurrent, *I*_ph_, at the optical beating frequency, *f*_1_–*f*_2_. The measured photoresponse values exhibit no roll-off up to 50 GHz, which is the frequency limitation of the utilized experimental setup. The dashed lines show the estimated frequency response for the graphene photodetector calculated from the device electrical model

A unique attribute of the graphene photodetector is that its superior bandwidth/responsivity performance is accompanied by an ultrafast photodetection speed. This ultrafast speed is made possible through the special design of the utilized gold-patched graphene nano-stripes, which offers broadband optical absorption in graphene and subpicosecond photocarrier transport times to the gold patches while maintaining low capacitive/resistive parasitics. A high-frequency electrical model of the graphene photodetector is shown in Fig. 4. The graphene resistance, *R*_g_, and SiO_2_ capacitance, *C*_ox_, are measured to be 70 Ω and 5.2 pF, respectively. Additionally, the gold-patch capacitance, *C*_g_, and substrate resistance, *R*_sub_, are estimated to be 12.9 fF and 5 MΩ, respectively. As can be observed from the device electrical model, the use of a high-resistivity silicon substrate leads to a large resistance, *R*_sub_, in series with the SiO_2_ capacitance, *C*_ox_, eliminating the negative impact of this capacitance on the ultrafast photodetection speed. Therefore, the photodetector frequency response predicted by this electrical model is dominated by the parasitic resistance of the graphene nano-stripes and parasitic capacitance of the gold patches, leading to a predicted photoresponse cutoff frequency of 425 GHz.

The high-frequency operation of the fabricated photodetector is characterized using two fiber-coupled, wavelength-tunable, distributed-feedback (DFB) lasers with 783 and 785 nm center wavelengths (TOPTICA #LD-0783-0080-DFB and #LD-0785-0080-DFB), as illustrated in Fig. 4. Both lasers have a spectral linewidth of 2 MHz and wavelength tunability range of 2.4 nm. When the two laser beams are combined in a single-mode fiber, they provide a tunable optical beating frequency ranging from 20 MHz to 2 THz. The combined laser beams are used to illuminate the graphene photodetector to induce a photocurrent at the optical beating frequency. A small portion of the optical beam is monitored with an optical spectrum analyzer to ensure that the two beating optical beams are maintained at the same power level. The photodetector output is probed using a ground-signal-ground (GSG) microwave probe (Picoprobe 50A-GSG-100-P-N) and monitored by a spectrum analyzer (HP8566B). A broadband bias-T (HP11612B) is used to apply the bias voltage and out-couple the high-frequency a.c. photocurrent. Figure 4 shows the photodetector output at various beating frequencies. As expected from the theoretical predictions, the experimental results exhibit no roll-off in the photodetector response of up to 50 GHz, which is the frequency limitation of the GSG probes and spectrum analyzer used. The variations in the photodetector output are due to fluctuations in the output power of the DFB lasers. To monitor the low-frequency operation of the fabricated photodetector, an optical chopper is used to modulate the beam emitted from one of the DFB lasers, with the photodetector output monitored by an oscilloscope (Supplementary Fig. S8). Despite using different experimental setups to characterize the frequency response of the fabricated photodetector, the high-frequency output power levels observed using the spectrum analyzer (Supplementary Fig. S9a) and low-frequency output voltage levels observed using the oscilloscope (Supplementary Fig. S9b) both indicate a responsivity of ~0.6 A/W at an input optical power of 1 μW, matching the photodetector DC responsivity.

## Conclusions

**Fig. 5:**
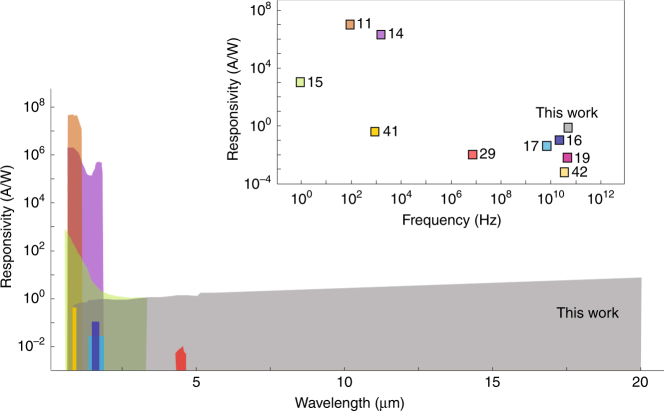
**Comparison of the responsivity, detection bandwidth, and operation speed for the fabricated room-temperature graphene photodetector with some of the highest-performance graphene photodetectors reported in the literature** ^11,14–17,19,29,41,42^

In this work, we present the unique electrical and optical characteristics of gold-patched graphene nano-stripes, which enable simultaneous broadband and ultrafast photodetection with high responsivity. We demonstrate high-responsivity photodetection from the visible to the infrared regime (0.6 A/W at 0.8 μm and 11.5 A/W at 20 μm), with operation speeds exceeding 50 GHz. Fig. 5 compares the specifications of the as-fabricated graphene photodetector based on gold-patched graphene nano-stripes with the specifications of some of the highest-performance room-temperature graphene photodetectors reported in the literature^11,14–17,19,29,41,42^. The presented graphene photodetector shows responsivity levels that are more than two orders of magnitude higher compared to previously reported high-speed graphene photodetectors^16,17,19^. The photodetector also shows response times that are more than seven orders of magnitude faster and bandwidths that are one order of magnitude broader compared to higher-responsivity graphene photodetectors based on quantum dots^11,14^ and tunneling barriers^15^. We expect the unique combination of broadband and ultrafast photodetection with high responsivity enabled by the gold-patched graphene nano-stripes will have a significant impact on future hyperspectral imaging and sensing systems. To further enhance the device performance, the symmetric gold patches can be replaced with asymmetric metal patches, which will lead to symmetry breaking for the electrical potential inside the graphene nano-stripes, enabling bias-free, low-dark-current device operation^43^.

## Electronic supplementary material

Supplementary Material

## Electronic supplementary material

**Supplementary information** accompanies for this paper at 10.1038/s41377-018-0020-2.
