# Supplementary material for: Gold-patched graphene nano-stripes for high-responsivity and ultrafast photodetection from the visible to infrared regime
Source: Light Sci Appl. 2018 Jun 20;7:20. doi: 10.1038/s41377-018-0020-2 (PMC6107021; doi:10.1038/s41377-018-0020-2)
Supplement: Supplementary file 1 — Supplementary Material [file 41377_2018_20_MOESM1_ESM.docx]

Gold-Patched Graphene Nano-Stripes for High-Responsivity and Ultrafast Photodetection from the Visible to Infrared Regime

Semih Cakmakyapan, Ping Keng Lu, Aryan Navabi, and Mona Jarrahi

Electrical Engineering Department, University of California Los Angeles, California 90095, USA

Correspondence: Mona Jarrahi, E-mail:[mjarrahi@ucla.edu](mailto:mjarrahi@ucla.edu)


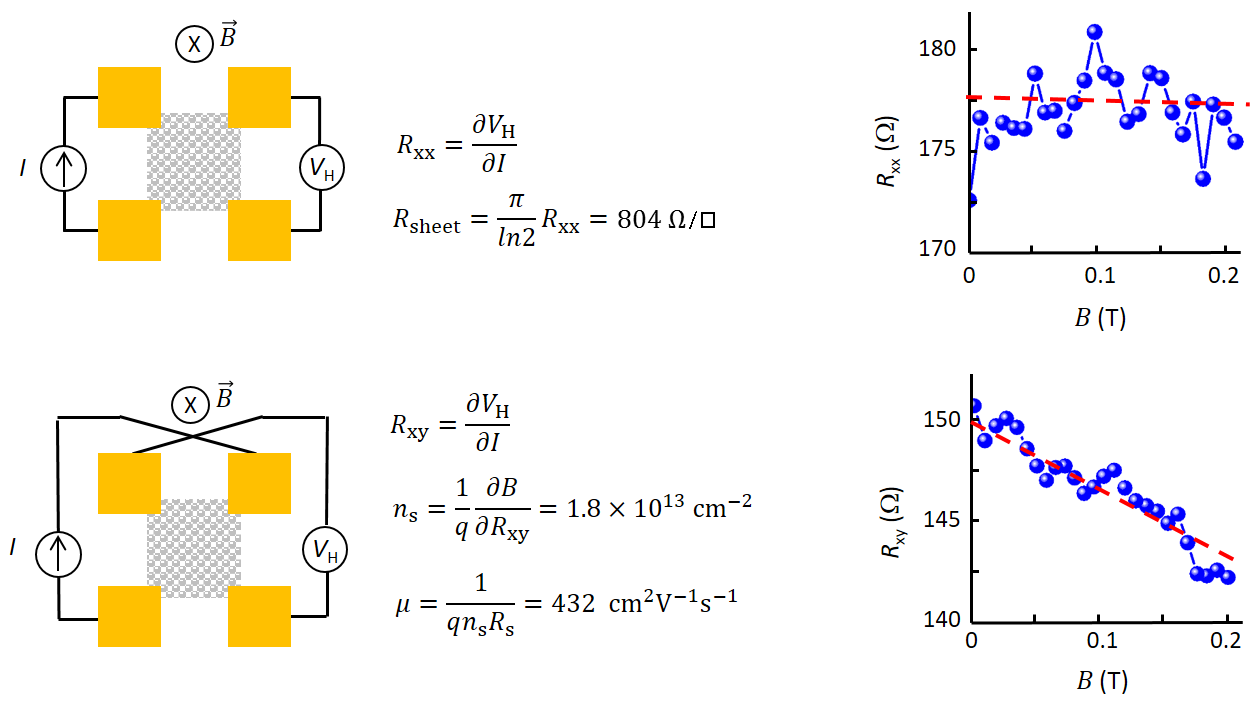


Fig. S1. Sheet resistance (*R*_sheet_), carrier concentration (*n*_s_), and mobility (*μ*) of the utilized monolayer graphene transferred to the thermal oxide layer, measured using the van der Pauw method. Four Ti/Au contacts are used to inject current, *I*, to the graphene sheet and measure the induced Hall voltage, *V*_H_, under an applied magnetic field, *B*.


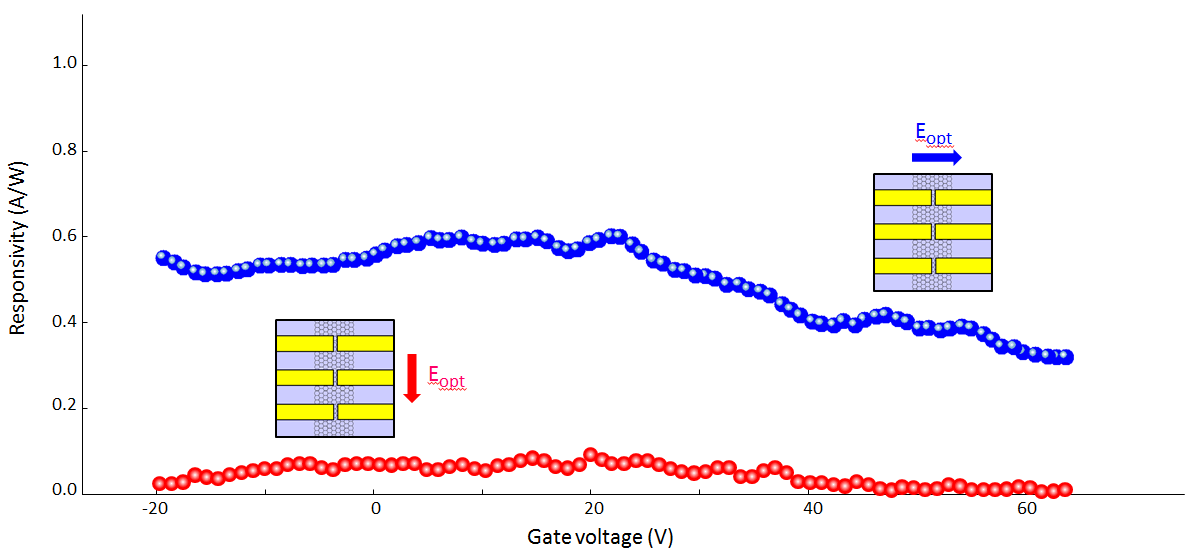


Fig. S2. Responsivity of the fabricated photodetector measured at an 800 nm wavelength under a bias voltage of 20 mV for an incident optical beam polarized normal to the graphene nano-stripes (blue data) and an incident optical beam polarized parallel with the graphene nano-stripes (red data). The responsivity data show strong polarization sensitivity due to the asymmetric geometry of the device. The strong polarization sensitivity of the presented photodetector could find many applications in polarimetric imaging and sensing systems.

1. (b)


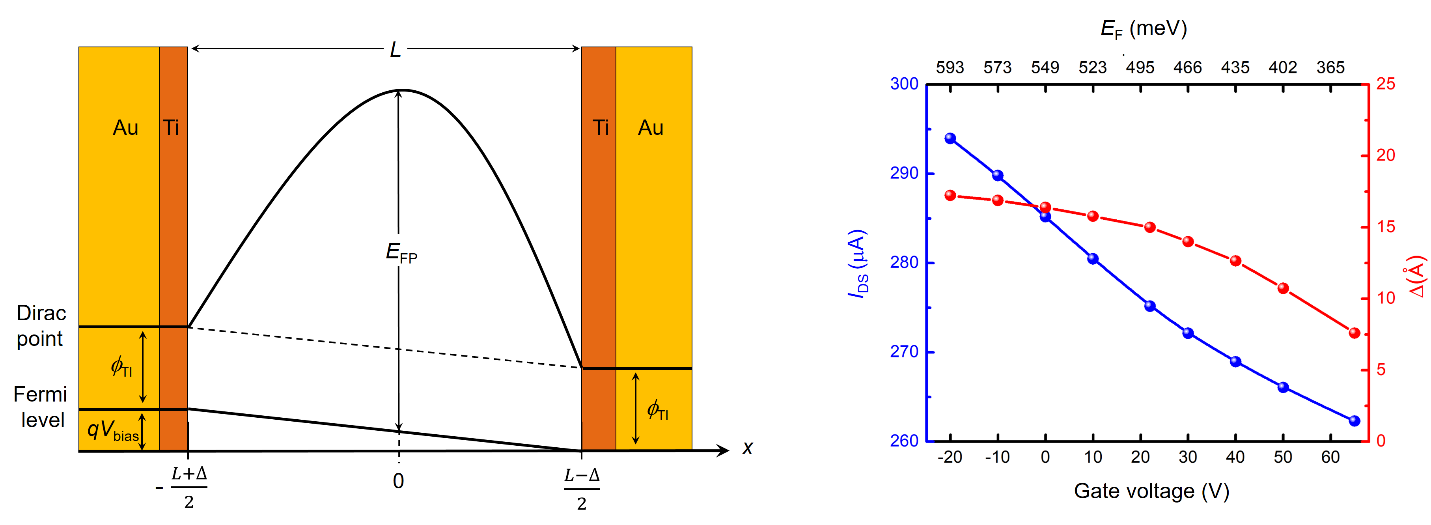


Fig. S3. (a) Band diagram of the gold-patched graphene nano-stripes: The gap between the two metal contacts to graphene (*L* = 50 nm), which is much shorter than the transition region at the metal-graphene interface, results in a significant band-bending that dominates the device performance. The Fermi energy level of graphene at the metal contacts (*φ*_Ti_ *=* 100 meV) is determined by the graphene doping induced by adsorption on the contact surface [1]. In order to draw the device band diagram, we assume that the peak Fermi energy level of graphene (*E*_FP_) between the metal contacts is determined by the graphene doping induced by the applied gate voltage. The validity of this assumption can be verified by the measured responsivity spectra shown in Fig. 2b. The band diagram is symmetric under a zero bias voltage with the peak Fermi energy level positioned in the middle of the gap. The potential gradient becomes steeper on the anode side under a non-zero bias voltage (*V*_bias_) and the peak Fermi energy point is slightly shifted to the anode side by Δ/2. Using a second-order (quadratic) approximation, the graphene Fermi energy level between the two metal contacts can be determined as:

$E_{F}\left( x \right)=\left\{ \begin{aligned} E_{\mathrm{FP}}-\left( \frac{2x}{L+\Delta} \right)^{2}\left[ E_{\mathrm{FP}}-qV_{\mathrm{bias}}-\varphi_{\mathrm{Ti}} \right] -\frac{L+\Delta}{2}<x<0 \\ \\ E_{\mathrm{FP}}-\left( \frac{2x}{L-\Delta} \right)^{2}\left[ E_{\mathrm{FP}}-\varphi_{\mathrm{Ti}} \right] 0<x<\frac{L-\Delta}{2} \end{aligned} \right.$

The device dark current, which is the difference between the current at the anode contact and cathode contact, for the 8 graphene nano-stripes used in the presented photodetector is given by:

$I_{\mathrm{DS}}=8I\left( \frac{L-\Delta}{2} \right)+8I\left( -\frac{L+\Delta}{2} \right)=8q\mu Wn\left( x \right)\times\frac{1}{q}\frac{\partial E_{F}\left( x \right)}{\partial x}|_{x = \frac{L-\Delta}{2}}+8q\mu Wn\left( x \right)\times\frac{1}{q}\frac{\partial E_{F}\left( x \right)}{\partial x}|_{x = -\frac{L+\Delta}{2}}$

where *q* is the electron charge, *W* is the width of the graphene nano-stripes, *μ* is carrier mobility, and *n*(*x*) is carrier density in graphene, $n\left( x \right)=\frac{1}{\pi\hbar^{2}{v^{2}}_{F}}{E^{2}}_{F}\left( x \right)$, where *v*_F_ is the Fermi velocity and *ħ* is the Planck constant divided by 2π. (b) The measured dark current of the fabricated graphene photodetector as a function of the gate voltage at an applied bias voltage of 20 mV (blue curve) and the band diagram parameters, *E*_FP_ and Δ, calculated from the measured dark current data at a bias voltage of 20 mV (red curve). For these calculations, we use the carrier mobility measured using the van der Pauw method (Fig. S1). The calculated parameters show a steeper potential gradient on the anode side at higher peak Fermi energy levels.

1. (b)


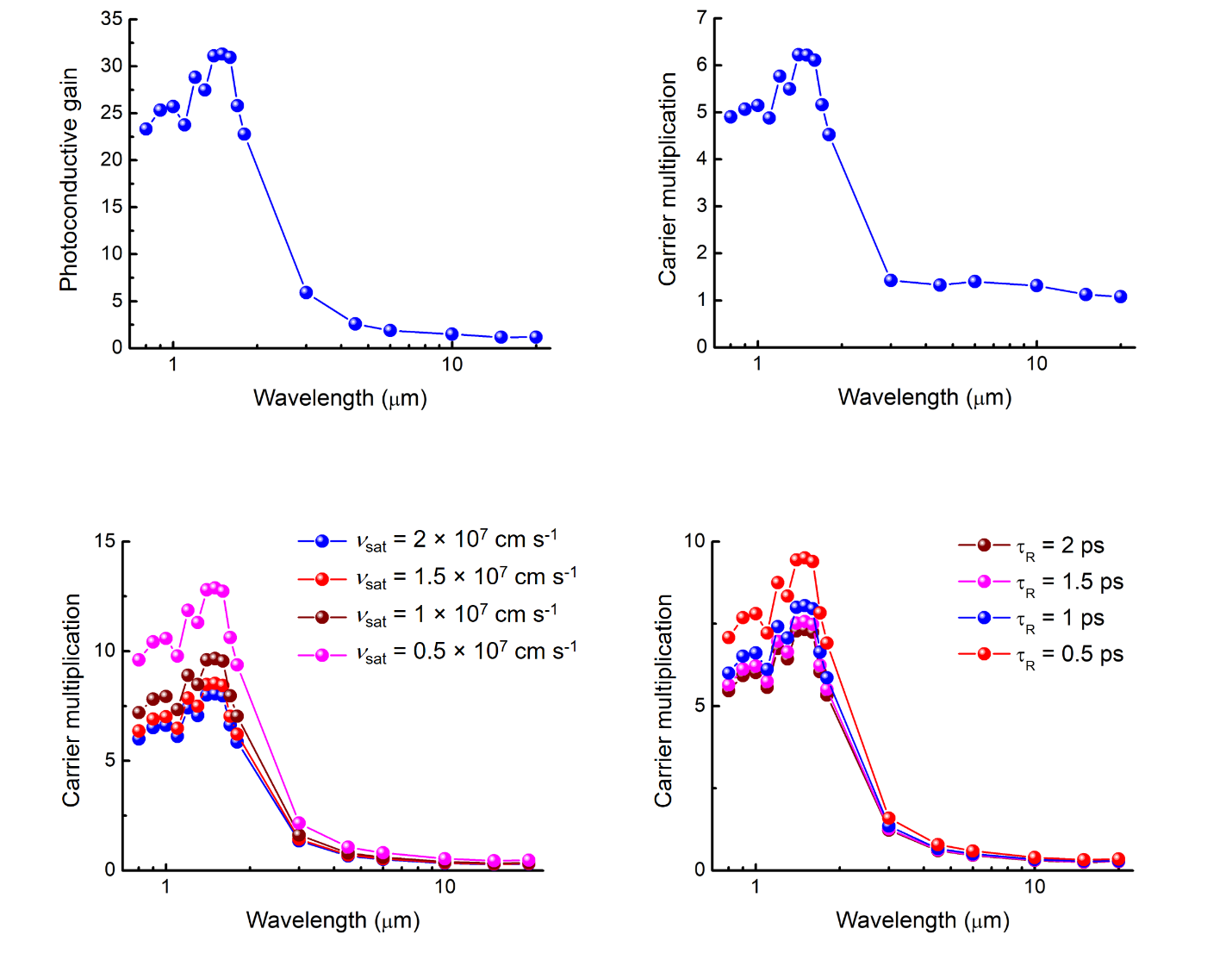


(d)

(c)

Fig. S4. (a) The estimated photoconductive gain of the fabricated photodetector at an optical power of 2.5 μW, gate voltage of 22 V, and bias voltage of 20 mV. When an optical beam is incident on the device, the photogenerated electrons and holes move according to the induced electric field given by:

$E\left( x \right)=\frac{1}{q}\frac{\partial E_{F}}{\partial x}$

Therefore, the photogenerated holes move to the center of the graphene nano-stripes where they eventually recombine, the photogenerated electrons in the $\frac{L-\Delta}{2}>x>$0 range move to the anode contact, and the photogenerated electrons in the $-\frac{L+\Delta}{2}<x<$0 range move to the cathode contact with a carrier drift velocity given by:

$v\left( x \right)={\mu\left| E\left( x \right) \right|}/\left[ 1+\frac{\mu\left| E\left( x \right) \right|}{v_{\mathrm{sat}}} \right]$

where *v*_sat_ is the carrier saturation velocity. The probability for an electron in the $\frac{L-\Delta}{2}>x>$0 range to reach the anode contact is given by $exp\left( -{t_{a}\left( x \right)}/{\tau_{R}} \right)$, where τ_R_ is the photocarrier recombination lifetime and $t_{a}\left( x \right)$ is the carrier transit time to the anode contact:

$t_{a}\left( x \right)=\int_{x}^{\frac{L-\Delta}{2}} \frac{dx}{v\left( x \right)}$

Therefore, the photocurrent flowing to the anode contact is calculated as:

$I_{ph-anode}= \left| \int_{0}^{\frac{L-\Delta}{2}} q\frac{\alpha P_{\mathrm{inc}}D_{p}\left( x \right)}{h\nu}\frac{M}{L}e^{- \frac{t_{a}\left( x \right)}{\tau_{R}}} dx \right|$

where *P*_inc_ is the incident optical power, $D_{P}\left( x \right)$ is the density of the absorbed optical power inside the graphene nano-stripes calculated from the Lumerical simulations shown in Fig. 1b, *α* is the optical absorption coefficient inside the gold-patched graphene nano-stripes, *hν* is photon energy, and *M* is the carrier multiplication factor. Similarly, the probability for an electron in the $-\frac{L+\Delta}{2}<x<$0 range to reach the cathode contact is given by $exp\left( -{t_{c}\left( x \right)}/{\tau_{R}} \right)$, where $t_{c}\left( x \right)$ is the carrier transit time to the cathode contact:

$t_{c}\left( x \right)=\int_{- \frac{L+\Delta}{2}}^{x} \frac{dx}{v\left( x \right)}$

Therefore, the photocurrent flowing to the cathode contact is calculated as:

$I_{ph-cathode}= \left| \int_{0}^{- \frac{L+\Delta}{2}} q\frac{\alpha P_{\mathrm{inc}}D_{p}\left( x \right)}{h\nu}\frac{M}{L}e^{- \frac{t_{c}\left( x \right)}{\tau_{R}}} dx \right|$

The induced photocurrent, which is the difference between the photocurrent flowing to the anode contact and cathode contact, is given by:

$I_{\mathrm{ph}}=I_{ph-anode}-I_{ph-cathode}=\left| \int_{0}^{\frac{L-\Delta}{2}} q\frac{\alpha P_{\mathrm{inc}}D_{p}\left( x \right)}{h\nu}\frac{M}{L}e^{- \frac{t_{a}\left( x \right)}{\tau_{R}}} dx \right|-\left| \int_{0}^{- \frac{L+\Delta}{2}} q\frac{\alpha P_{\mathrm{inc}}D_{p}\left( x \right)}{h\nu}\frac{M}{L}e^{- \frac{t_{c}\left( x \right)}{\tau_{R}}} dx \right|$

By using the calculated photoconductive gain, *G*, from the measured photocurrent, $I_{\mathrm{ph}}=qG\frac{\alpha P_{\mathrm{inc}}}{h\nu}$, and assuming a uniform carrier multiplication factor inside the graphene nano-stripes, the carrier multiplication factor is calculated as a function of the optical wavelength.

$M=\frac{G}{\frac{1}{L}\left| \int_{0}^{\frac{L-\Delta}{2}} D_{p}\left( x \right)e^{- \frac{t_{a}\left( x \right)}{\tau_{R}}} dx \right|- \frac{1}{L}\left| \int_{0}^{- \frac{L+\Delta}{2}} {D_{p}\left( x \right)e}^{- \frac{t_{c}\left( x \right)}{\tau_{R}}} dx \right|}$

(b) The estimated carrier multiplication factor at an optical power of 2.5 μW, gate voltage of 22 V, and bias voltage of 20 mV for a carrier saturation velocity of 2×10^7^ cm s^-1^ [2] and a photocarrier recombination lifetime of 1 ps [3-5] as a function of the optical wavelength. The results indicate carrier multiplication factors larger than 1 in the visible and near-infrared frequencies (higher carrier multiplication factors are estimated for smaller carrier saturation velocity and shorter carrier lifetime values as shown in (c) and (d), respectively). There are two possible mechanisms for carrier multiplication. The first mechanism involves an interband scattering process (also called impact ionization) in which an excited electron in the conduction band relaxes to a lower energy state, and the released energy is transferred for the excitation of another electron from the valance band to the conduction band. This mechanism increases the carrier density within the conduction band and is suppressed by the Pauli blocking. Therefore, increasing graphene doping reduces its efficiency [6-8]. The second mechanism involves a Coulomb-induced intraband scattering process (also called hot carrier multiplication) in which an excited electron relaxes to a lower energy state, and the released energy is transferred for the excitation of another electron below the Fermi energy level to a state above the Fermi energy level. This mechanism becomes more efficient at higher doping levels due to the availability of a larger number/density of states that electrons can be excited to [6, 7, 9]. Considering the relatively high carrier concentration levels in the graphene nano-stripes, the observed carrier multiplication in the presented graphene photodetector is anticipated to be governed by the second mechanism, the Coulomb-induced intraband scattering process. As expected, higher carrier multiplication factors are achieved in the visible and near-infrared wavelengths compared to the infrared wavelengths. The reason is that the photogenerated electrons in response to the visible and near-infrared beams are excited to higher energy levels in the conduction band. As a result, they give rise to the excitation of a larger number of secondary electrons by transferring more energy during relaxation. Also, lower carrier multiplication factors are achieved at higher optical power levels. This decrease is due to the increase in the carrier recombination rate at high photogenerated carrier densities, which reduces the carrier scattering time and the carrier multiplication efficiency [8-10].

As it can be observed from the above discussions, only the photovoltaic (PV) response of the gold-patched graphene nano-stripes has been considered to estimate the carrier multiplication factor in graphene. Our calculations show that the photothermoelectric (PTE) effect does not play a significant role in the photo-response of the presented photodetector due to the geometric symmetry of the gold-patched graphene nano-stripes, which offers a relatively symmetric profile of the density of states and temperature for the PTE-induced photocurrents flowing from graphene to the metal contacts. Including the PTE-induced photocurrent in our calculations would only slightly increase the estimated carrier multiplication factor. This is because the direction of the PTE-induced photocurrent in the presented photodetector is in the opposite direction to the PV-induced photocurrent. The reason is that hot carriers diffuse from regions with lower density of states to regions with higher density of states to maximize the entropy. Since the Fermi energy level has a slightly steeper slope on the anode junction than the cathode junction, the PTE-induced electron current flowing from the anode junction (lower density of states) to the graphene nano-stripes (higher density of states) to maximize the entropy would be larger than the PTE-induced electron current flowing from the cathode junction (lower density of states) to the graphene nano-stripes (higher density of states) to maximize the entropy.


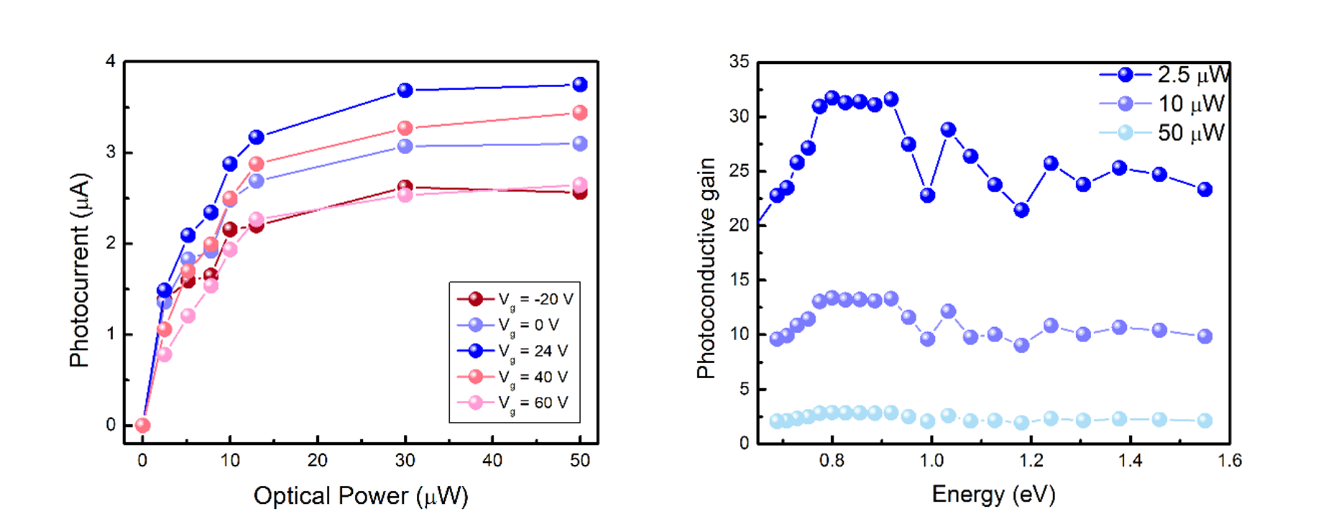


1. (b)

Fig. S5. (a) Output photocurrent of the fabricated graphene photodetector at an 800 nm wavelength as a function of the incident optical power and gate voltage. (b) Photoconductive gain of the fabricated graphene photodetector in the visible and near-infrared wavelength ranges as a function of the incident optical power. All of the measurements are performed at an applied bias voltage of 20 mV.


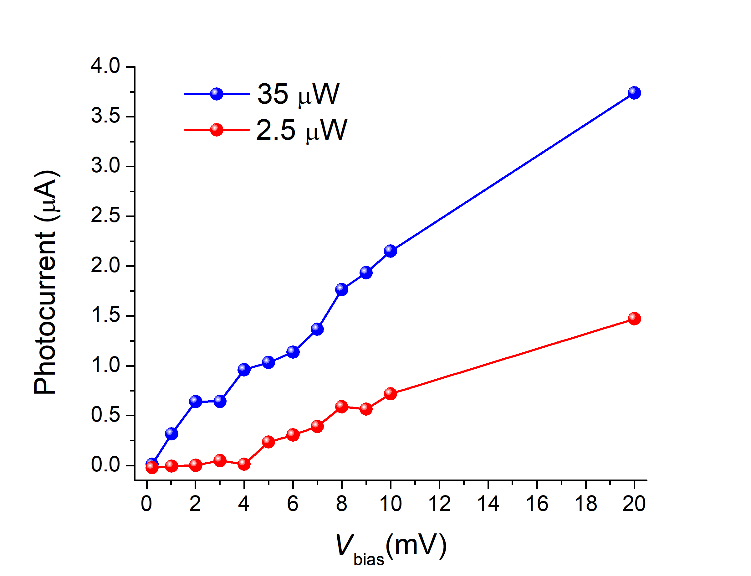


Fig. S6. Output photocurrent of the fabricated graphene photodetector at an 800 nm wavelength as a function of the applied bias voltage for an optical power of 2.5 μW (red data) and 35 μW (blue data).


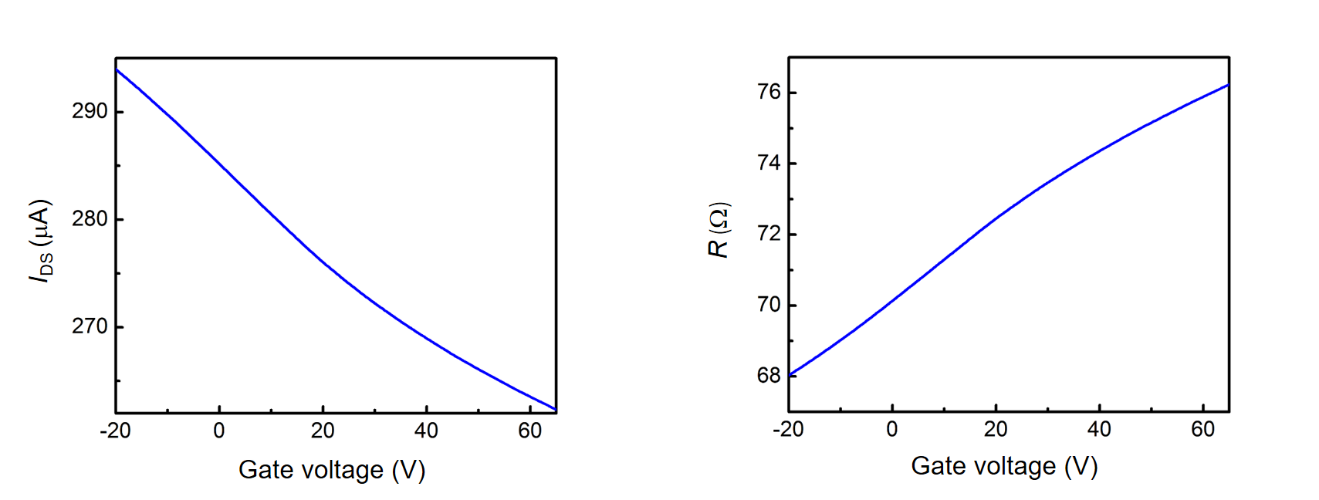


(a) (b)

Fig. S7. (a) Dark current of the fabricated graphene photodetector as a function of the gate voltage at an applied bias voltage of 20 mV. (b) Resistance of the fabricated graphene photodetector as a function of the gate voltage.


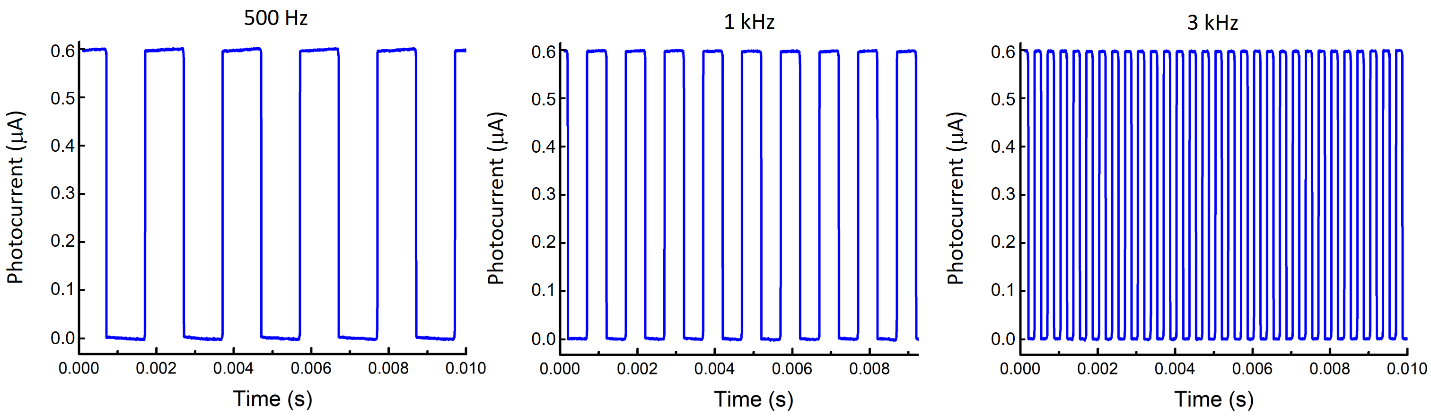


Fig. S8. Time-domain photodetector output photocurrent in response to an incident optical beam modulated at 500 Hz, 1 kHz, and 3 kHz.

(a)


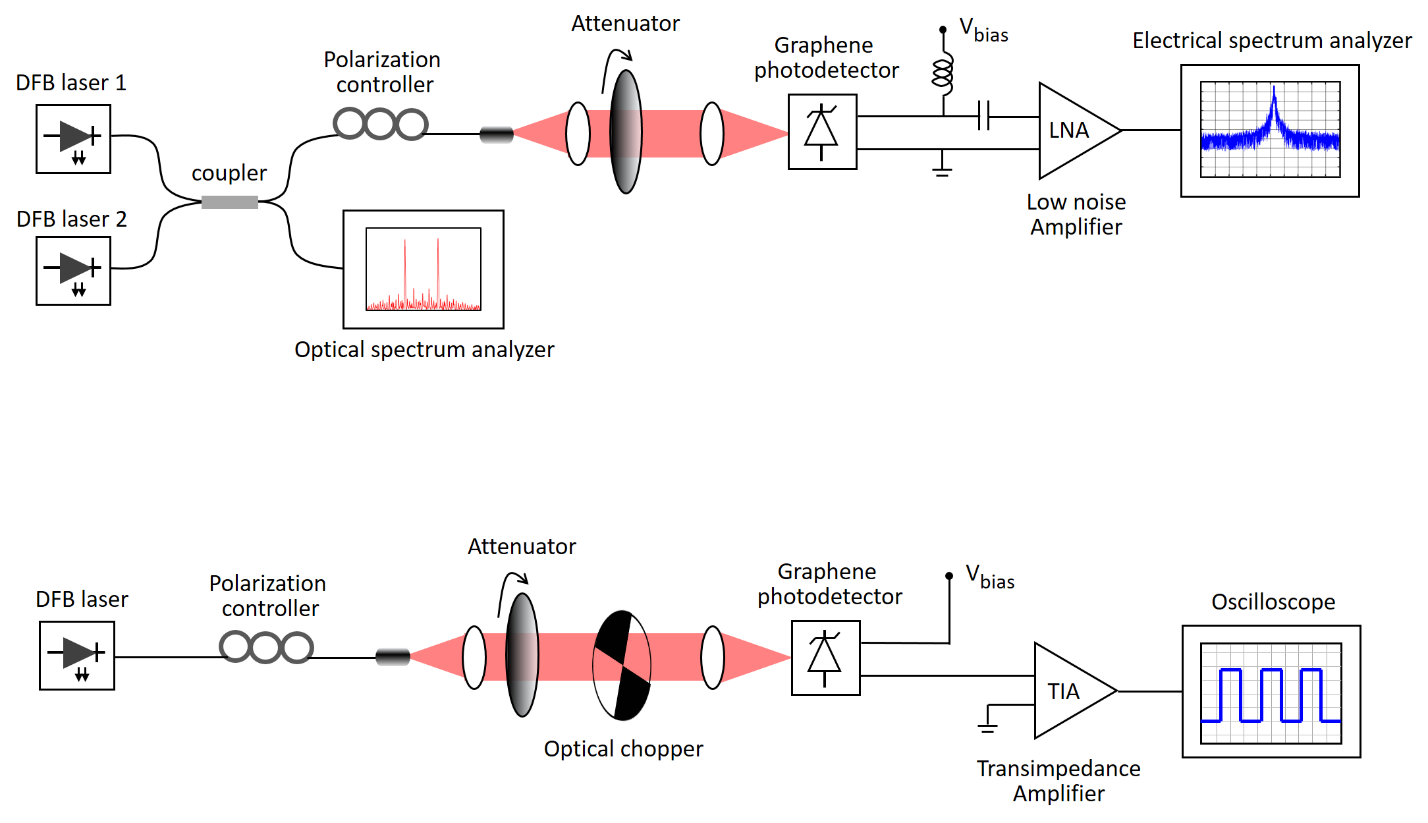


(b)

Fig. S9. Experimental setups for characterizing photodetector frequency response at high frequencies and low frequencies are illustrated in (a) and (b), respectively.

| Model number | Center wavelength | Power transmission | Filter bandwidth |
| --- | --- | --- | --- |
| Thorlabs FB3000-500 | 3 μm | 1.08 % | 0.48 μm |
| Thorlabs FB4500-500 | 4.5 μm | 0.98 % | 0.51 μm |
| Thorlabs FB6000-500 | 6 μm | 0.79 % | 0.38 μm |
| Spectrogon BBP9900-11000 | 10 μm | 0.56 % | 1.7 μm |
| Thorlabs FB19M15 | 15 μm | 0.94 % | 3.1 μm |
| Thorlabs FB19M20 | 20 μm | 1.06 % | 2.8 μm |

Table S1. Characteristics of the utilized infrared filters for the infrared responsivity measurements.

References

1. Xia, F., Mueller, T., Golizadeh-Mojarad, R., Freitag, M., Lin, Y. M., Tsang, J., Perebeinos, V. & Avouris, P. Photocurrent imaging and efficient photon detection in a graphene transistor. Nano Lett. 9, 3, 1039-1044 (2009).
2. Dorgan, V. E., Bae, M. H. & Pop, E. Mobility and saturation velocity in graphene on SiO_2_. Appl. Phys. Lett. 97, 8 082112 (2010).
3. Rana, F., George, P. A., Strait, J. H., Dawlaty, J., Shivaraman, S., Chandrashekhar, M. & Spencer, M. G. Carrier recombination and generation rates for intravalley and intervalley phonon scattering in graphene. Phys. Rev. B 79, 115447 (2009).
4. George, P. A., Strait, J. H., Dawlaty, J., Shivaraman, S., Chandrashekhar, M, Rana, F., & Spencer, M. Ultrafast optical-pump terahertz-probe spectroscopy of the carrier relaxation and recombination dynamics in epitaxial graphene. Nano Lett. 8, 4248–4251 (2008).
5. Vasko, F. T. & Ryzhii, V. Voltage and temperature dependencies of conductivity in gated graphene. Phys. Rev. B 76, 233404 (2007).
6. Kadi, F., Winzer, T., Knorr, A. & Malic, E. Impact of doping on the carrier dynamics in graphene. Sci. Rep. 5, 16841 (2015).
7. Malic, E., Winzer, T., Wendler, F. & Knorr, A. Review on carrier multiplication in graphene. Phys. Status Solidi B 253, 2303–2310 (2016).
8. Plotzing, T., Winzer, T., Malic, E., Neumaier, D., Knorr, A. & Kurz, H. Experimental verification of carrier multiplication in graphene. Nano Lett. 14, 5371-5375 (2014).
9. Tielrooij, K. J., Song, J. C. W., Jensen, S. A., Centeno, A., Pesquera, A., Elorza, A. Z., Bonn, M., Levitov, L. S. & Koppens, F. H. L. Photoexcitation cascade and multiple hot-carrier generation in graphene. Nat. Phys. 9, 248−252 (2013).
10. Brida, D., Tomadin, A., Manzoni, C., Kim, Y. J., Lombardo, A., Milana, S., Nair, R. R., Novoselov, K. S., Ferrari, A. C., Cerullo, G. & Polini, M. Ultrafast collinear scattering and carrier multiplication in graphene. Nat. Comm. 4, 1987 (2013).
